# Drug-Target Interaction Prediction through Label Propagation with Linear Neighborhood Information

**DOI:** 10.3390/molecules22122056

**Published:** 2017-11-25

**Authors:** Wen Zhang, Yanlin Chen, Dingfang Li

**Affiliations:** 1 School of Computer, Wuhan University, Wuhan 430072, China; zhangwen@whu.edu.cn; 2 School of Mathematics and Statistics, Wuhan University, Wuhan 430072, China; chenyanlin@whu.edu.cn

**Keywords:** drug-target interactions, label propagation, linear neighborhood, integrated information

## Abstract

Interactions between drugs and target proteins provide important information for the drug discovery. Currently, experiments identified only a small number of drug-target interactions. Therefore, the development of computational methods for drug-target interaction prediction is an urgent task of theoretical interest and practical significance. In this paper, we propose a label propagation method with linear neighborhood information (LPLNI) for predicting unobserved drug-target interactions. Firstly, we calculate drug-drug linear neighborhood similarity in the feature spaces, by considering how to reconstruct data points from neighbors. Then, we take similarities as the manifold of drugs, and assume the manifold unchanged in the interaction space. At last, we predict unobserved interactions between known drugs and targets by using drug-drug linear neighborhood similarity and known drug-target interactions. The experiments show that LPLNI can utilize only known drug-target interactions to make high-accuracy predictions on four benchmark datasets. Furthermore, we consider incorporating chemical structures into LPLNI models. Experimental results demonstrate that the model with integrated information (LPLNI-II) can produce improved performances, better than other state-of-the-art methods. The known drug-target interactions are an important information source for computational predictions. The usefulness of the proposed method is demonstrated by cross validation and the case study.

## 1. Introduction

The identification of potential drug-target interactions is a crucial task in drug discovery, which helps to find novel targets for existing drugs or identify targets for new drugs [1]. Wet experiments are reliable ways of determining interactions between drugs and targets, but they are cost-intensive and time-consuming [2]. In contrast, computational methods provide economic and efficient alternative to predict possible drug-target interactions with high reliability for further experiments.

To the best of our knowledge, researchers collect drug-target interaction data, and construct the public databases. Available drug-target data facilitate the development of drug-target interaction prediction methods. Traditional computational methods include molecular docking simulation methods and ligand-based methods. Though docking simulation methods are effective, they cannot work without three-dimensional (3D) structures of targets [3]. Ligand-based methods perform well when there are sufficient known ligands for a target protein, but such methods are not suitable for large-scale data [4]. 

In addition, several methods have been proposed based on properties of drug and targets. Kuhn et al. [5] used molecular features and target proteins to predict drug-target relations. Garcia-Sosa et al. [6,7] introduced logistic regression and naïve Bayesian classifiers for classification of compounds into one disease category or organ by studying target-ligand data. Cao et al. [8] found that genes that have spatial interactions may have similar molecular function and developed a new gene function prediction method based on gene-gene interacting networks. Xu et al. [9] proposed a stochastic gradient boosting algorithm to predict effective drug combination. Zeng et al. [10] developed a novel features fusion method and adopted the random forest classifier for protein-protein interaction prediction. Wei et al. utilized the random forest classifier [11] and an ensemble classifier called LibD3C [12] to predict protein-protein interaction.

Recently, a great number of machine learning methods have been introduced for the drug-target interaction prediction, and machine learning-based methods are roughly divided into four categories: classification methods, matrix factorization methods, kernel methods and network inference methods. Classification methods take drug-target interaction pairs and non-interaction pairs as positive instances or negative instances, and build the classification models for predictions. For example, Nagamine et al. [13] and Wang et al. [14] constructed support vector machine (SVM) models; Tabei et al. [15] utilized logistic regression and SVM. Matrix factorization methods use the matrix factorization technique to reconstruct drug-target interactions. The kernelized Bayesian matrix factorization with twin kernels (KBMF2K) [16] and multiple similarity collaborative matrix factorization (MSCMF) [17] have been used for predictions and graph-regularized matrix factorization (GRMF) [18]. Kernel methods include the pair kernel method (PKM) [19], net Laplacian regularized least squares (NetLapRLS) [20], and regularized least squares with Kronecker product kernel (RLS-Kron) [21]. Network inference methods formulate the drug-target interactions as the graph learning. Bleakley and Yamanishi [22] built bipartite local model (BLM). Mei et al. [23] improved the BLM by considering new drug candidates through its neighbors’ interaction profiles. Chen et al. [24] applied a random walk technique to walk on a drug-drug similarity network, a target-target similarity network and known drug-target interaction networks for predictions. Cheng et al. [25] adopted the resource allocation method to infer interactions in the drug-target bipartite network. Moreover, there are different types of machine learning-based methods [26,27,28,29,30].

Drug-drug similarity or target-target similarity are critical components in many drug-target interaction prediction methods [17,19,20,21,22,23,24]. How to define the similar drugs (targets) is critical, and the point is to calculate drug-drug similarity. To the best of our knowledge, there are different ways of calculating drug-drug similarity based on feature vectors, such as cosine similarity, Gauss similarity and Jaccard similarity. Cosine similarity consists in measuring the cosine of the angle between two vectors in an inner product space. Gauss similarity utilizes the Gauss kernel function to measure the similarity. Jaccard similarity considers the interaction of components and the union.

In this paper, we propose a label propagation method with linear neighborhood information (LPLNI) for drug-target interaction predictions. Firstly, we calculate drug-drug linear neighborhood similarity in the feature spaces by considering how to reconstruct data points from neighbors. Then, we take similarities as the manifold of drugs and assume the manifold unchanged in the interaction space. At last, we predict unobserved interactions between known drugs and targets by using drug-drug linear neighborhood similarity and known drug-target interactions. We present a feature of drugs named the interaction profile from the known drug-target interactions. The LPLNI model, based on the interaction profiles, perform well in the computational experiments, achieving AUPR up to 0.9051, 0.9461, 0.9658, and 0.9464 on the enzymes (Es) dataset, the GPCRs dataset, the ion channels (ICs) dataset, and the nuclear receptors (NRs) dataset, respectively. Further, we incorporate drug structure information into the LPLNI model by a nonlinear strategy, improving AUPR to 0.9069, 0.9469, 0.9684, and 0.9492 on the Es dataset, the GPCRs dataset, the ICs dataset, and the NRs dataset, respectively. The experimental results show that our method outperforms other state-of-the-art methods on these four benchmark datasets.

## 2. Results and Discussion

### 2.1. Evaluation Metrics

In order to evaluate the performances of prediction models, computational experiments were conducted on four benchmark datasets. Here, we adopted leave-one-out cross validation (LOOCV) to test model performances. That is, each drug-target pair was left out in turn, and remaining pairs were used as the training set to build models for predictions. We repeated the procedure until each drug-target pair is ever tested.

The AUC and AUPR are the most popular evaluation metrics in the previous works. AUC is the area under the receiver operating characteristic (ROC) curve, which plots the true positive rate (TPR) versus the false positive rate (FPR). AUPR is the area under the precision-recall curve, which plots the ratio of true positives among the predicted positives for each recall rate. There are more negative instances than positive ones, and AUPR punishes the false positives more in evaluation [31]. Therefore, we adopted AUPR as the primary metric and used AUC to evaluate models.

### 2.2. The Performances of the LPLNI Models

In this section, we evaluate the performances of the LPLNI models. Since we had the interaction profiles and fingerprints for drugs, we respectively used these features to calculate the linear neighborhood similarities and then built LPLNI models. Here, we used the Pubchem fingerprint for analysis.

There are two parameters K and α in LPLNI, in which K is the number of neighbors in the linear neighborhood similarity (LNS), and α is the probability of absorbing target information from neighbors. These parameters may influence the results, and we can build LPLNI models using different parameter values. The number of drug neighbors K should be less than the number of all drugs, and the four benchmark datasets, i.e., the nuclear receptors (NRs) dataset, the G-protein coupled receptors (GPCRs) dataset, the ion channels (ICs) dataset, and the enzymes (Es) dataset, contain 54, 223, 210, and 445 drugs, respectively. Therefore, we considered different neighborhood numbers K 10, 30, and 50 for the NRs dataset, 60, 120, and 180 for the GPCRs and ICs datasets, and 120, 240, and 360 for the Es dataset. In addition, absorbing probability α should be greater than zero, and smaller than one. Hence, for parameter α we chose values from 0.1 to 0.9 (with a step size of 0.1).

The drug-drug similarity is critical for LPLNI. To demonstrate the superiority of linear neighborhood similarity, we also considered cosine similarity, Jaccard similarity, and Gauss similarity and applied label propagation to build similarity-based prediction models. The Gauss function calculates the similarity by $\mathrm{Gauss}\left(x_{i},x_{j}\right)=\exp\left(-\|x_{i}-x_{j}\|{}^{2}/\sigma \right)$, which has the bandwidth parameter σ, and we set $\sigma =\sum_{i}\left|x_{i}\right|/n_{d}$ as in [23], where $x_{i}$ is the feature vector of the i-th drug, and $n_{d}$ is the number of drugs.

**Figure 1:**
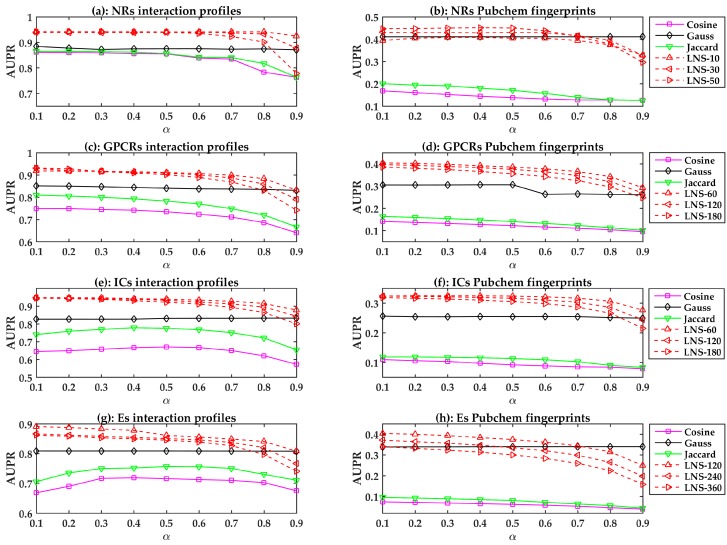
The area under the precision-recall curve (AUPR) values of the similarity-based models with different parameters. LNS-10 means LN similarity-based models constructed with 10 neighbors. Other symbols have the similar meanings.

All prediction models are evaluated using LOOCV. The performances of different similarity-based models are shown in Figure 1. In general, the linear neighborhood similarity can lead to better performances than can cosine similarity, Gauss similarity, or Jaccard similarity. The possible reason for the superior performances of the LPLNI models is that the linear neighborhood similarity describes the linear relationship of data points in the feature space. The linear neighborhood similarity is then smoothly transferred into the interaction space, and LPLNI utilizes the label propagation to make predictions based on the same linear relationship of data points in the interaction space.

Moreover, we observed that the LPLNI models based on the interaction profiles have better performances than the LPLNI models based on the Pubchem fingerprint, which indicates that the interaction profiles are an information source of utmost importance for prediction.

### 2.3. The Performances of LPLNI Models with Integrated Information

In machine learning, combining diverse information of drugs can improve the performance of prediction models [32,33,34,35,36,37]. In Section 2.2, our study demonstrates that only the use of interaction profiles of drugs can lead to high-accuracy prediction models; however, we still attempted to incorporate structural information of drugs to further improve accuracy.

Since we had nine different fingerprints, we firstly built individual LPLNI models based on different fingerprint features and evaluated their usefulness. The leave-one-out cross validation performances of the prediction models are shown in Table 1. Among all fingerprints, Daylight, Extended and Hybridization fingerprints produce better performances than others on the benchmark datasets. Although the performances of fingerprints are lower than the interaction profiles, fingerprints can still provide information for the drug-target interaction predictions. According to their performances, Daylight fingerprints, Extended fingerprints, and Hybridization fingerprints were adopted to incorporate into the interaction profile-based models.

By using the strategy described in Section 3.4, we incorporated the three fingerprints into the interaction profile-based model and developed the prediction model with integrated information, named “LPLNI-II.” As shown in Table 1, LPLNI-II can produce better results than individual feature-based models on the benchmark datasets, improving the AUPR values of 0.9464 to 0.9492 and AUC values of 0.9532 to 0.9919 (on NRs dataset), indicating the usefulness of combing various information of drugs.

### 2.4. Comparison with State-of-the-Art Methods

To the best of our knowledge, a great number of methods were proposed to predict drug-target interactions. NetLapRLS [20] trained two classifiers based on the chemical and genomic information with the interaction profiles separately, and then linearly combined the two classifiers to develop the prediction model. RLS-Kron [21] considered chemical structures, genomic sequences, and the interaction profiles, then calculated the similarity by the Gaussian function, and utilized the Regularized Least Squares (RLS) classifier to build prediction models. The model based on the interaction profiles could produce high-accuracy performances, and the final prediction model was developed by integrating diverse information with the Kronecker product. These methods and our method utilize the interaction profiles as the primary information sources to develop prediction models. To demonstrate the superiority of our method, we adopted NetLapRLS and RLS-Kron for comparison. All methods were evaluated by leave-one-out cross validation (LOOCV).

Since RLS-Kron and our method can make high-accuracy predictions using only the interaction profiles, we firstly built prediction models based on the interaction profiles and compared their performances. As shown in Table 2, the AUPR values of LPLNI are 0.9051, 0.9461, 0.9658 and 0.9464, higher than RLS-Kron on the enzymes (Es) dataset, the G-protein coupled receptors (GPCRs) dataset, the ion channels (ICs) dataset, and the nuclear receptors (NRs) dataset, respectively. In addition, LPLNI produces superior AUC performances on the GPCRs dataset, the ICs dataset, and the NRs dataset. Therefore, the interaction profile-based LNLPI model produces better results than the interaction profile-based RLS-Kron model on these benchmark datasets.

Further, we tested the performances of the LPLNI model with integrated information (LPLNI-II) by comparing LPLNI-II with RLS-Kron and NetLapRLS. As shown in Table 3, LPLNI-II can outperform benchmark methods on the GPCRs dataset, ICs dataset, and NRs dataset. Therefore, the LPLNI-II can integrate different information and make high-accuracy predictions.

### 2.5. Case Study

To test the potential of LNLPI in the drug-target interaction predictions, we built models based on known interactions of the Es dataset and then made predictions for unknown interactions. We checked the top 10 interactions predicted by our method and looked for evidences in SuperTarget [38] to support our discoveries. SuperTarget contains updating interactions from several drug databases, i.e., DrugBank, KEGG, etc. As shown in Table 4, 4 predictions out of 10 are confirmed, and results indicate that our method is capable of predicting novel interactions.

## 3. Materials and Methods

### 3.1. Datasets

There are several databases that provide information about drugs and drug-target interactions and that can be used for predicting unobserved drug-target interactions.

The Pubchem database [39,40] can provide chemical structures. The DrugBank database [41,42,43,44] is a comprehensive bioinformatics resource that includes targets, transporters, and enzymes of drugs. The KEGG database [45,46] is a collection of protein pathways that are associated with drug targets. BRENDA [47] is a comprehensive collection of enzyme and metabolic data, and is updated by extracting information from primary literature. SuperTarget [38] contains more than 2500 target proteins, which are annotated with about 7300 relations to 1500 drugs.

To study potential drug-target interactions, we used four benchmark datasets of drug-target interactions, which were compiled by Yamanishi et al. [48]. There are mainly four types of target proteins: enzymes (Es), ion channels (ICs), G-protein coupled receptors (GPCRs), and nuclear receptors (NRs). In Yamanishi’s datasets, the drug-target interactions were classified into four subsets, which are associated with different types of targets. Table 5 lists the details of the four datasets.

### 3.2. Features

**Figure 2:**
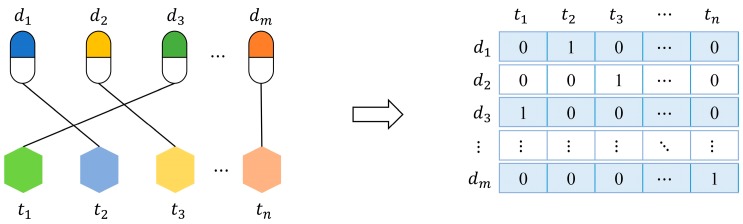
A drug-target interaction network and interaction profiles of drugs.

In order to build prediction models, we should represent drugs or targets as feature vectors. Firstly, we present a feature named “interaction profile” for drugs (targets) from known interactions. As shown in Figure 2, let $\left\{d_{1},d_{2},\cdots ,d_{m}\right\}$ be a set of given drugs, $\left\{t_{1},t_{2},\cdots ,t_{n}\right\}$ be a set of given targets, and their interactions can be formalized as an interaction network. The interaction profile of a drug (target) is a binary vector describing the presence or absence of interaction with every target (drug) in the network.

Since we collect drug structures from KEGG DRUG, we also represent drugs as feature vectors based on their substructures. Structural features of drugs are well known as fingerprints, which are bit vectors with elements indicating the frequencies or the existence of certain substructures. As listed in Table 6, there are different drug fingerprints, and we adopt Chemical Development Kit (CDK) [49] to calculate these fingerprints and then use them as structural feature vectors.

### 3.3. The Label Propagation Method with Linear Neighborhood Information

In this section, we introduce the label propagation method with linear neighborhood information (LPLNI), which has two steps: calculation of linear neighborhood similarity and label propagation-based prediction.

Let us introduce several notations. Given $n_{d}$ drugs and $n_{t}$ targets, their interactions are organized as an interaction matrix $Y=\left(Y_{1},Y_{2},\cdots ,Y_{n_{t}}\right)\in {\mathbb{R}}^{n_{d}\times n_{t}}$, where $Y_{i}$ is the interaction profile of the i-th target. $1=y_{ij}\in Y$ if the i-th drug interacts with the j-th target, else, $y_{ij}=0$. Each drug can be represented by a p-dimension feature vector $x_{i}$ (for example, the interaction profile), $i=1,2,\;\cdots ,n_{d}$.

#### 3.3.1. Linear Neighborhood Similarity

Roweis et al. [50] revealed that a data point and its neighbors are close to a locally linear patch of the manifold, and Wang et al. [51] discovered that each point can be optimally reconstructed by its neighbors. Based on these studies [50,51], we calculated the drug-drug similarity by considering how to reconstruct the data point through its neighbors, as per our previous work [52].

Here, we represent drugs as feature vectors $x_{i}$, $i=1,2,\;\cdots ,n_{d}$ and take them as data points in the feature space. We reconstruct each data point $x_{i}$ by linear combination of its neighbors and formulate the optimization problem as follows:

(1) $$\underset{\omega_{i}}{\min}\varepsilon_{i}=\|x_{i}-\sum_{i_{j}:x_{i_{j}}\in N\left(x_{i}\right)}\omega_{i,i_{j}}x_{i_{j}}{\|}^{2}=\sum_{i_{j},i_{k}:X_{i_{j}},X_{i_{k}}\in N\left(X_{i}\right)}\omega_{i,i_{j}}G_{i_{j},i_{k}}^{i}\omega_{i,i_{k}}=\omega_{i}^{T}G^{i}\omega_{i}$$

$$s.t.\;\sum_{i_{j}:x_{i_{j}}\in N\left(x_{i}\right)}\omega_{i,i_{j}}=1,\;\omega_{i,i_{j}}\geq 0,j=1,\cdots ,K$$

where ‖·‖ is the Euclidean norm, and $N\left(x_{i}\right)$ represents the set of K $\left(0<K<n_{d}\right)$ nearest neighbors (by Euclidean distance) of $x_{i}$. $\omega_{i}={\left(\omega_{i,i_{1}}\cdots ,\omega_{i,i_{K}}\right)}^{T}$ and $G_{i_{j},i_{k}}^{i}={\left(x_{i}-x_{i_{j}}\right)}^{T}\left(x_{i}-x_{i_{k}}\right)$ is the entry of the Gram matrix $G^{i}\in {\mathbb{R}}^{K\times K}$. $\omega_{i,i_{j}}$ represents the weights of $x_{i_{j}}$ for reconstructing $x_{i}$ and can be considered as the similarity between $x_{i}$ and $x_{i_{j}}$. Clearly, $\omega_{i,j}=0$ if $x_{j}\notin N\left(x_{i}\right)$.

We notice that the matrix $G^{i}$ is likely to be singular if the K neighbors are close to each other. In this case, it is hard to obtain the unique solution of the optimization problem. In order to avoid the singular matrix and enhance generalization capability, we introduce regularization for the reconstructive weights and present the optimization problem:

(2) $$\underset{\omega_{i}}{\min}\;\omega_{i}^{T}G^{i}\omega_{i}+\lambda_{i}\|\omega_{i}{\|}^{2}$$

$$s.t.\;e^{T}\omega_{i}=1,\;\omega_{i}\geq 0$$

where $\lambda_{i}$ is the regularization parameter and column vector $e={\left(1,1,\cdots ,1\right)}^{T}$. 

The parameter $\lambda_{i}$ controls the relative value between reconstruction error $\omega_{i}^{T}G^{i}\omega_{i}$ and the regularization term $\|\omega_{i}{\|}^{2}$. Since spectral norm is compatible and Gram matrix $G^{i}$ is symmetric and positive semidefinite, we have

(3) $$\omega_{i}^{T}G^{i}\omega_{i}=\|{\left(G^{i}\right)}^{\frac{1}{2}}\omega_{i}{\|}^{2}\leq \|{\left(G^{i}\right)}^{\frac{1}{2}}{\|}^{2}\|\omega_{i}{\|}^{2}=\rho \left(G^{i}\right)\|\omega_{i}{\|}^{2}$$

where $\rho \left(G^{i}\right)$ is spectral radius of $G^{i}$. Here, we can estimate value range of $\omega_{i}^{T}G^{i}\omega_{i}$ and $\|\omega_{i}{\|}^{2}$. Therefore, we can roughly set

(4) $$\lambda_{i}=\varepsilon \rho \left(G^{i}\right)$$

in the practical use, and ε is a small number satisfying ε≪1. We set ε to 0.01 for simplicity.

**Figure 3:**
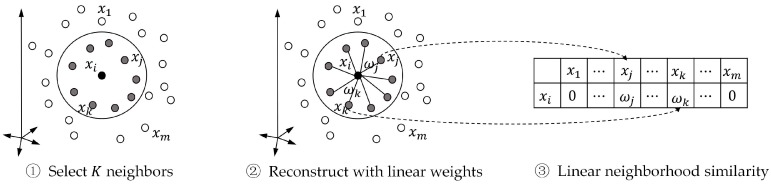
Procedure of calculating linear neighborhood similarity.

We can use the standard quadratic programing to solve Equation (2), and its solutions is named the “linear neighborhood similarity” (LNS). We calculate the weights for data points, and concentrate them row by row, and form the similarity matrix $W\in {\mathbb{R}}^{n_{d}\times n_{d}}$. The entire procedure of calculating LNS is summarized in Figure 3.

#### 3.3.2. Label Propagation

Based on the drug-drug similarity, we formulate a directed graph, which uses drugs as nodes and similarities $W\left(i,j\right)=\omega_{ij}$ as weights. It is worth mentioning that usually $\omega_{ij}\neq \omega_{ji}$.

In the graph, the known interactions of drugs with given targets are taken as the initial label information of nodes, and the label information is then updated. In the update, a node absorbs label information for its neighbors with the probability α∈(0, 1) and retains the initially label information with the probability 1−α. The update process for the i-th label of nodes at the k-th iteration is written as

(5) $$F_{i}^{\left(k\right)}=\alpha WF_{i}^{\left(k-1\right)}+\left(1-\alpha \right)Y_{i}$$

where $Y_{i}$ is the i-th column vector of the interaction matrix Y (i.e., the i-th initial labels for all nodes). Further, we can formulate the update for all target labels in matrix form:

(6) $$F^{\left(k\right)}=\alpha WF^{\left(k-1\right)}+\left(1-\alpha \right)Y$$

where $F^{\left(k\right)}\in {\mathbb{R}}^{n_{d}\times n_{t}}$ represents that label matrix in the kth iteration, and $F^{\left(0\right)}=Y$. We will analyze the convergence of this iterative process Equation (6) in Theorem 1.

**Theorem** **1.** *The iterative process, Equation (6), will converge to a solution F, that is*

(7) $$F=\left(1-\alpha \right){\left(I-\alpha W\right)}^{-1}Y$$

*where* $I\in {\mathbb{R}}^{n_{d}\times n_{d}}$ *is the identity matrix.*

**Proof of Theorem** **1.** Note that $F^{\left(0\right)}=Y$, the iterative process Equation (6) can be rewritten as follows

$$F^{\left(k\right)}=\alpha WF^{\left(k-1\right)}+\left(1-\alpha \right)Y={\left(\alpha W\right)}^{2}F^{\left(k-2\right)}+\left(1-\alpha \right)\left(I+\alpha W\right)Y=\cdots \;={\left(\alpha W\right)}^{k}Y+\left(1-\alpha \right)\overset{k-1}{\underset{i=0}{\sum }}{\left(\alpha W\right)}^{i}Y.$$

Since the spectral radius of W or ρ(W)≤1 and 0<α<1, then

$$\underset{k\to \infty }{\lim}{\left(\alpha W\right)}^{k}=0,\;\underset{k\to \infty }{\lim}\sum_{i=0}^{k-1}{\left(\alpha W\right)}^{i}={\left(I-\alpha W\right)}^{-1}.$$

Therefore,

$$F=\underset{k\to \infty }{\lim}F^{\left(k\right)}=\left(1-\alpha \right){\left(I-\alpha W\right)}^{-1}Y.$$

$F\in \propto^{n_{d}\times n_{t}}$ is the final label matrix, presenting the predicted scores for drug-target pairs.

### 3.4. LPLNI with Integrated Information

In this paper, we consider the interaction profile feature of drugs and targets and consider different fingerprint features of drugs. Therefore, we can calculate different similarities based on different features and then build different prediction models. Generally, combining diverse models can enhance predictive performances [53,54,55,56].

Here, we consider a nonlinear strategy to integrate different prediction models. Given n models, they will produce n predicted scores for a drug-target pair, denoted as $F=\left\{F^{1},F^{2},\cdots ,F^{n}\right\}$, and the integrated score is given by the following binomial logistic regression model in the conditional probability form:

(8) $$P\left(Y_{ij}=1|F\right)=\frac{exp\left(\sum_{k}\alpha_{k}F_{ij}^{k}+b\right)}{1+exp\left(\sum_{k}\alpha_{k}F_{ij}^{k}+b\right)}$$

where $\alpha_{k}\in \propto$, k=1,2,⋯,n, and b∈R. The parameters are estimated by maximum likelihood estimation based on known interactions and their predicted scores. 

In the prediction stage, the predicted scores from the n models are aggregated by Equation (8) to produce the final predictions. 

We abbreviate the LPLNI model with integrated information as “LPLNI-II”.

## 4. Conclusions

In this paper, we propose a drug-target interaction prediction method with linear neighborhood information, and the method can utilize known interactions to make high-accuracy predictions. Further, we incorporated structural information into the prediction models to improve performances. Computational experiments show that our method outperforms other state-of-the-art methods on the benchmark datasets. The potential of the method is also validated in the case study. In conclusion, the proposed method is a promising tool for drug-target interaction prediction.

## Figures and Tables

**Table 1:** Performances of label propagation method with linear neighborhood information (LPLNI) models and LPLNI-II models on the four datasets.

Features	Methods	NRs	ICs	GPCRs	Es
Daylight	LPLNI	**0.4519**	**0.3326**	**0.4254**	**0.4094**
		**0.7868**	**0.7605**	**0.8771**	**0.8307**
EState	LPLNI	0.2958	0.2437	0.3096	0.2770
		0.6903	0.7098	0.8480	0.8055
Extended	LPLNI	**0.4452**	**0.3382**	**0.4317**	**0.4153**
		**0.7820**	**0.7741**	**0.8783**	**0.8261**
GraphOnly	LPLNI	0.3177	0.3226	0.3525	0.3507
		0.7478	0.7606	0.8483	0.7939
Hybridization	LPLNI	**0.4226**	**0.3462**	**0.4047**	**0.4050**
		**0.8001**	**0.7962**	**0.8747**	**0.8224**
Klekota-Roth	LPLNI	**0.4665**	0.3030	0.3819	0.3360
		**0.8103**	0.7355	0.8580	0.8179
MACCS	LPLNI	0.3764	**0.3400**	0.3881	0.3804
		0.7712	**0.7543**	0.8621	0.8360
Pubchem	LPLNI	0.4470	0.3234	**0.4038**	**0.4039**
		0.7561	0.7522	**0.8822**	**0.8405**
Substructure	LPLNI	0.3202	0.3092	0.2942	0.2875
		0.7539	0.7662	0.8465	0.8068
Interaction profile	LPLNI	0.9464	0.9658	0.9461	0.9051
		0.9532	0.9890	0.9683	0.9465
Day&Ext&Hyb&Int	LPLNI-II	0.9492	0.9684	0.9469	0.9069
		0.9919	0.9947	0.9769	0.9700

The value of each fingerprint represents AUPR values (previous row) and area under the receiver operating characteristic (ROC) curve (AUC) values (next row). The bold type indicates the top 4 in terms of AUC and AUPR values. Day&Ext&Hyb&Int: using Daylight, Extended, Hybridization, and the interaction profile as features.

**Table 2:** Performances of LPLNI and RLS-Kron based on the interaction profiles.

Datasets	Features	Methods	AUC	AUPR
Es	Interaction profile	RLS-Kron	**0.9830**	0.8850
		LPLNI	0.9465	**0.9051**
GPCRs	Interaction profile	RLS-Kron	0.9470	0.7130
		LPLNI	**0.9683**	**0.9461**
ICs	Interaction profile	RLS-Kron	0.9860	0.9270
		LPLNI	**0.9890**	**0.9658**
NRs	Interaction profile	RLS-Kron	0.9060	0.6100
		LPLNI	**0.9532**	**0.9464**

The bold type indicates the highest AUC/AUPR values. The following tables maintain uniform standards.

**Table 3:** Performances of LPLNI-II and other state-of-the-art methods.

Datasets	Features	Methods	AUC	AUPR
Es	chem&gen&int	RLS-Kron	0.9780	**0.9150**
	chem&gen&int	NetLapRLS	**0.9830**	N.A.
	chem&int	LPLNI-II	0.9700	0.9069
GPCRs	chem&gen&int	RLS-Kron	0.9540	0.7130
	chem&gen&int	NetLapRLS	0.9710	N.A.
	chem&int	LPLNI-II	**0.9769**	**0.9469**
ICs	chem&gen&int	RLS-Kron	0.9840	0.9430
	chem&gen&int	NetLapRLS	0.9860	0.N.A.
	chem&int	LPLNI-II	**0.9947**	**0.9684**
NRs	chem&gen&int	RLS-Kron	0.9220	0.6840
	chem&gen&int	NetLapRLS	0.8880	0.N.A.
	chem&int	LPLNI-II	**0.9919**	**0.9492**

N.A.: not available. chem, gen, and int are abbreviations for chemical structure, genomic sequence, and the interaction profile, respectively.

**Table 4:** The top 10 new predicted interactions on the Es dataset.

Rank	Pair	Description	Confirmed?
1	D00574	Aminoglutethimide (USP/INN)	
	hsa1589	cytochrome P450, family 21, subfamily A, polypeptide 2	
2	D00437	Nifedipine (JP15/USP/INN)	Yes
	hsa1559	cytochrome P450, family 2, subfamily C, polypeptide 9	
3	D00542	Halothane (JP15/USP/INN)	Yes
	hsa1571	cytochrome P450, family 2, subfamily E, polypeptide 1	
4	D00410	Metyrapone (JP15/USP/INN)	
	hsa1583	cytochrome P450, family 11, subfamily A, polypeptide 1	
5	D00139	Methoxsalen (JP15/USP)	Yes
	hsa1543	cytochrome P450, family 1, subfamily A, polypeptide 1	
6	D00437	Nifedipine (JP15/USP/INN)	
	hsa1585	cytochrome P450, family 11, subfamily B, polypeptide 2	
7	D00691	Diprophylline (JAN/INN)	
	hsa8654	phosphodiesterase 5A, cGMP-specific	
8	D00691	Diprophylline (JAN/INN)	
	hsa5152	phosphodiesterase 9A	
9	D00691	Diprophylline (JAN/INN)	Yes
	hsa5150	phosphodiesterase 7A	
10	D00691	Diprophylline (JAN/INN)	
	hsa50940	Peptidyl-prolyl cis-trans isomerase A	

**Table 5:** Statistics of four drug-target interaction datasets.

Datasets	$n_{d}$	$n_{t}$	$E_{dt}$	$\bar{n_{d}}$	$\bar{n_{t}}$	Sparsity
Es	445	664	2926	6.5753	4.4066	0.0099
GPCRs	223	95	635	2.8475	6.6842	0.0299
ICs	210	204	1476	7.0286	7.2353	0.0345
NRs	54	26	90	1.6667	3.4615	0.0641

$n_{d}$ is the number of drugs, $n_{t}$ is the number of targets, $E_{dt}$ is the number of known interactions, $\bar{n_{d}}$ is the average number of targets for each drug, and $\bar{n_{t}}$ is the average number of drugs for each target. Sparsity is known interactions divided by all possible interaction pairs.

**Table 6:** Descriptions of nine fingerprints.

Fingerprints	Descriptions
Daylight	Daylight fingerprints based on hashing molecular subgraphs
EState	This fingerprinter generates 79 bit fingerprints using the E-State fragments
Extended	These fingerprints extends the CDK with additional bits describing ring features
Graph Only	Specialized version of the CDK Fingerprinter that does not take bond orders into account
Hybridization	This fingerprinter takes into account SP2 hybridization states
Klekota-Roth	This fingerprinter presence of 4860 substructures
MACCS	This fingerprinter generates 166 bit MACCS keys.
Pubchem	These fingerprints are of the structural key type, of length 881
Substructure	The fingerprint currently supports 307 substructures

## References

[1] Ding H., Takigawa I., Mamitsuka H., Zhu S. (2013). Similarity-based machine learning methods for predicting drug-target interactions: A brief review. Brief. Bioinform..

[2] Whitebread S., Hamon J., Bojanic D., Urban L., Hamon J. (2005). In vitro safety pharmacology profiling: An essential tool for successful drug development. Drug Discov. Today.

[3] Ballesteros J., Palczewski K. (2001). G protein-coupled receptor drug discovery: Implications from the crystal structure of rhodopsin. Curr. Opin. Drug Discov. Dev..

[4] Hansch C., Maloney P.P., Fujita T., Muir R.M. (1962). Correlation of biological activity of phenoxyacetic acids with hammett substituent constants and partition coefficients. Nature.

[5] Kuhn M., Campillos M., Gonzalez P., Jensen L.J., Bork P. (2008). Large-scale prediction of drug-target relationships. FEBS Lett..

[6] Garcia-Sosa A.T., Oja M., Hetenyi C., Maran U. (2012). Druglogit: Logistic discrimination between drugs and nondrugs including disease-specificity by assigning probabilities based on molecular properties. J. Chem. Inf. Model..

[7] Garcia-Sosa A.T., Maran U. (2013). Drugs, non-drugs, and disease category specificity: Organ effects by ligand pharmacology. SAR QSAR Environ. Res..

[8] Cao R.Z., Cheng J.L. (2015). Deciphering the association between gene function and spatial gene-gene interactions in 3D human genome conformation. BMC Genom..

[9] Xu Q., Xiong Y., Dai H., Kumari K.M., Xu Q., Ou H.Y., Wei D.Q. (2017). PDC-SGB: Prediction of effective drug combinations using a stochastic gradient boosting algorithm. J. Theor. Biol..

[10] Zeng J.C., Li D.P., Wu Y.F., Zou Q., Liu X.R. (2016). An empirical study of features fusion techniques for protein–protein interaction prediction. Curr. Bioinform..

[11] Wei L.Y., Zou Q., Liao M.H., Lu H.J., Zhao Y.M. (2016). A novel machine learning method for cytokine-receptor interaction prediction. Comb. Chem. High Throughput Screen..

[12] Wei L., Xing P., Zeng J., Chen J., Su R., Guo F. (2017). Improved prediction of protein–protein interactions using novel negative samples, features, and an ensemble classifier. Artif. Intell. Med..

[13] Nagamine N., Sakakibara Y. (2007). Statistical prediction of protein chemical interactions based on chemical structure and mass spectrometry data. Bioinformatics.

[14] Wang Y.C., Yang Z.X., Wang Y., Deng N.Y. (2010). Computationally probing drug-protein interactions via support vector machine. Lett. Drug Des. Discov..

[15] Tabei Y., Pauwels E., Stoven V., Takemoto K., Yamanishi Y. (2012). Identification of chemogenomic features from drug-target interaction networks using interpretable classifiers. Bioinformatics.

[16] Gonen M. (2012). Predicting drug-target interactions from chemical and genomic kernels using bayesian matrix factorization. Bioinformatics.

[17] Zheng X., Ding H., Mamitsuka H., Zhu S. (2013). Collaborative matrix factorization with multiple similarities for predicting drug-target interactions. Proceedings of the 19th ACM SIGKDD International Conference on Knowledge Discovery and Data Mining.

[18] Ezzat A., Zhao P.L., Wu M., Li X.L., Kwoh C.K. (2017). Drug-target interaction prediction with graph regularized matrix factorization. IEEE ACM Trans. Comput. Bioinform..

[19] Jacob L., Vert J.P. (2008). Protein-ligand interaction prediction: An improved chemogenomics approach. Bioinformatics.

[20] Xia Z., Wu L.Y., Zhou X., Wong S.T. (2010). Semi-supervised drug-protein interaction prediction from heterogeneous biological spaces. BMC Syst. Biol..

[21] Van Laarhoven T., Nabuurs S.B., Marchiori E. (2011). Gaussian interaction profile kernels for predicting drug-target interaction. Bioinformatics.

[22] Bleakley K., Yamanishi Y. (2009). Supervised prediction of drug-target interactions using bipartite local models. Bioinformatics.

[23] Mei J.P., Kwoh C.K., Yang P., Li X.L., Zheng J. (2013). Drug-target interaction prediction by learning from local information and neighbors. Bioinformatics.

[24] Chen X., Liu M.X., Yan G.Y. (2012). Drug-target interaction prediction by random walk on the heterogeneous network. Mol. Biosyst..

[25] Cheng F., Liu C., Jiang J., Lu W., Li W., Liu G., Zhou W., Huang J., Tang Y. (2012). Prediction of drug-target interactions and drug repositioning via network-based inference. PLoS Comput. Biol..

[26] Ezzat A., Wu M., Li X.L., Kwoh C.K. (2017). Drug-target interaction prediction using ensemble learning and dimensionality reduction. Methods.

[27] Kuang Q.F., Li Y.Z., Wu Y.M., Li R., Dong Y.C., Li Y., Xiong Q., Huang Z.Y., Li M.L. (2017). A kernel matrix dimension reduction method for predicting drug-target interaction. Chemom. Intell. Lab..

[28] Buza K., Peska L. (2017). Drug-target interaction prediction with bipartite local models and hubness-aware regression. Neurocomputing.

[29] Ding Y.J., Tang J.J., Guo F. (2017). Identification of drug-target interactions via multiple information integration. Inf. Sci..

[30] Peska L., Buza K., Koller J. (2017). Drug-target interaction prediction: A bayesian ranking approach. Comput. Methods Programs Biomed..

[31] Davis J., Goadrich M. (2006). The relationship between precision-recall and roc curves. Proceedings of the 23rd International Conference on Machine Learning.

[32] Zhang W., Chen Y.L., Tu S.K., Liu F., Qu Q.L. Drug side effect prediction through linear neighborhoods and multiple data source integration. Proceedings of the 2016 IEEE International Conference on Bioinformatics and Biomedicine.

[33] Zhang W., Chen Y.L., Liu F., Luo F., Tian G., Li X.H. (2017). Predicting potential drug-drug interactions by integrating chemical, biological, phenotypic and network data. BMC Bioinform..

[34] Zhang W., Zhu X.P., Fu Y., Tsuji J., Weng Z.P. The prediction of human splicing branchpoints by multi-label learning. Proceedings of the 2016 IEEE International Conference on Bioinformatics and Biomedicine.

[35] Li D.F., Luo L.Q., Zhang W., Liu F., Luo F. (2016). A genetic algorithm-based weighted ensemble method for predicting transposon-derived pirnas. BMC Bioinform..

[36] Luo L.Q., Li D.F., Zhang W., Tu S.K., Zhu X.P., Tian G. (2016). Accurate prediction of transposon-derived pirnas by integrating various sequential and physicochemical features. PLoS ONE.

[37] Zhang W., Liu F., Luo L.Q., Zhang J.X. (2015). Predicting drug side effects by multi-label learning and ensemble learning. BMC Bioinform..

[38] Gunther S., Kuhn M., Dunkel M., Campillos M., Senger C., Petsalaki E., Ahmed J., Urdiales E.G., Gewiess A., Jensen L.J. (2008). Supertarget and matador: Resources for exploring drug-target relationships. Nucleic Acids Res..

[39] Wang Y., Xiao J., Suzek T.O., Zhang J., Wang J., Bryant S.H. (2009). Pubchem: A public information system for analyzing bioactivities of small molecules. Nucleic Acids Res..

[40] Li Q.L., Chen T.J., Wang Y.L., Bryant S.H. (2010). Pubchem as a public resource for drug discovery. Drug Discov. Today.

[41] Wishart D.S., Knox C., Guo A.C., Shrivastava S., Hassanali M., Stothard P., Chang Z., Woolsey J. (2006). Drugbank: A comprehensive resource for in silico drug discovery and exploration. Nucleic Acids Res..

[42] Wishart D.S., Knox C., Guo A.C., Cheng D., Shrivastava S., Tzur D., Gautam B., Hassanali M. (2008). Drugbank: A knowledgebase for drugs, drug actions and drug targets. Nucleic Acids Res..

[43] Knox C., Law V., Jewison T., Liu P., Ly S., Frolkis A., Pon A., Banco K., Mak C., Neveu V. (2011). Drugbank 3.0: A comprehensive resource for ‘omics’ research on drugs. Nucleic Acids Res..

[44] Law V., Knox C., Djoumbou Y., Jewison T., Guo A.C., Liu Y.F., Maciejewski A., Arndt D., Wilson M., Neveu V. (2014). Drugbank 4.0: Shedding new light on drug metabolism. Nucleic Acids Res..

[45] Kanehisa M., Goto S., Hattori M., Aoki-Kinoshita K.F., Itoh M., Kawashima S., Katayama T., Araki M., Hirakawa M. (2006). From genomics to chemical genomics: New developments in kegg. Nucleic Acids Res..

[46] Kanehisa M., Goto S., Furumichi M., Tanabe M., Hirakawa M. (2010). Kegg for representation and analysis of molecular networks involving diseases and drugs. Nucleic Acids Res..

[47] Schomburg I., Chang A., Ebeling C., Gremse M., Heldt C., Huhn G., Schomburg D. (2004). Brenda, the enzyme database: Updates and major new developments. Nucleic Acids Res..

[48] Yamanishi Y., Araki M., Gutteridge A., Honda W., Kanehisa M. (2008). Prediction of drug-target interaction networks from the integration of chemical and genomic spaces. Bioinformatics.

[49] Steinbeck C., Hoppe C., Kuhn S., Floris M., Guha R., Willighagen E.L. (2006). Recent developments of the (cdk)—An open-source java library for chemo- and bioinformatics. Curr. Pharm. Des..

[50] Roweis S.T., Saul L.K. (2000). Nonlinear dimensionality reduction by locally linear embedding. Science.

[51] Wang F., Zhang C.S. (2008). Label propagation through linear neighborhoods. IEEE Trans. Knowl. Data Eng..

[52] Zhang W., Qu Q., Zhang Y., Wang W. (2018). The linear neighborhood propagation method for predicting long non-coding RNA–protein interactions. Neurocomputing.

[53] Zhang W., Zou H., Luo L.Q., Liu Q.C., Wu W.J., Xiao W.Y. (2016). Predicting potential side effects of drugs by recommender methods and ensemble learning. Neurocomputing.

[54] Zhang W., Niu Y.Q., Zou H., Luo L.Q., Liu Q.C., Wu W.J. (2015). Accurate prediction of immunogenic T-cell epitopes from epitope sequences using the genetic algorithm-based ensemble learning. PLoS ONE.

[55] Zhang W., Niu Y.Q., Xiong Y., Zhao M., Yu R.W., Liu J. (2012). Computational prediction of conformational B-cell epitopes from antigen primary structures by ensemble learning. PLoS ONE.

[56] Zhang W., Liu J., Zhao M., Li Q.J. (2012). Predicting linear B-cell epitopes by using sequence-derived structural and physicochemical features. Int. J. Data Min. Bioinform..

